# Supplementary material for: Elevated body temperature exacerbates arrhythmia and seizure-like activity in a zebrafish model of Timothy syndrome
Source: bioRxiv. 2025 Mar 14:2025.03.11.642683. Preprint. [Version 1] doi: 10.1101/2025.03.11.642683 (PMC12190319; doi:10.1101/2025.03.11.642683)
Supplement: Supplement 5 [file NIHPP2025.03.11.642683v1-supplement-5.pdf]

bioRxiv preprint doi: https://doi.org/10.1101/2025.03.11.642683; this version posted March 14, 2025. The copyright holder for this preprint (which was not certified by peer review) is the author/funder. This article is a US Government work. It is not subject to copyright under 17 USC 105 and is also made available for use under a CC0 license.

Supplementary figure 1. Cacna1c expression during brain development in zebrafish

Expression of *cacna1c* during embryonic and early larval brain development (0-120 hpf) compared to markers of neuronal maturation. Data is mean read counts per stage, using cells annotated as neural in DanioCell, smoothed and normalized to maximum expression for each gene.

Supplementary figure 2. Characterization of TS2 zebrafish

A. Survival by genotype for sibling larvae raised in mixed groups of 20–30 individuals. N = 3 groups per genotype. * Tukey's test, $p = 0.001$.

B. Length of adult fish by genotype and sex (m, male ; f, female). * t-test, $p < 0.05$.

C-D. Normalized junction counts from RNA-seq for exon 7 to either exon 8 or 8A, and exon 8 or 8A to exon 9 in wild-type, heterozygous and mutant TS2 larvae, at 2 dpf (C) and 6 dpf (D). Between-group differences not significant for any junction.

Supplementary figure 3. Differentially expressed genes in TS2 mutants

A. Chromosomal location of differentially expressed genes in TS2 mutant larvae compared to wild-type siblings (blue markers). Location of *cacna1c* indicated (orange marker).

B. Volcano plot for genes differentially expressed between wild-type and mutant TS2 in 2 dpf larvae using RNA-seq of anterior tissue section (head and heart).

C. Change in expression for four genes that were significantly different between heterozygous and wild-type siblings larvae, that were also nominally different in the wild-type/homozygote comparison.

Supplementary figure 4. Ventricular bradycardia and antisense control of exon 8 splicing

A. Contraction frequency (beats per minute, bpm) in sibling wild-type, heterozygous and homozygous mutant larvae. Atrial frequency was significantly reduced in mutants (*, t-test $p = 0.0036$) but not in

bioRxiv preprint doi: https://doi.org/10.1101/2025.03.11.642683; this version posted March 14, 2025. The copyright holder for this preprint (which was not certified by peer review) is the author/funder. This article is a US Government work. It is not subject to copyright under 17 USC 105 and is also made available for use under a CC0 license.

heterozygous larvae. For ventricle frequency, because of outlier individuals, we used a Mann-Whitney test to compare: #, $p = 0.016$.

B. RT-PCR and restriction digest for 2 dpf embryos injected with control morpholino (c), 1 ng per embryo i7e8 morpholino (1) or 3 ng i7e8 morpholino (3).

C-D. Meta-analysis of three experiments injecting morpholinos into wild-type (C) and mutant (D) embryos. Standard mean difference and confidence interval for atrial contraction frequency between control and i7e8 morphants is shown for each experiment, with diamonds showing overall effect, which was significant in mutants (standard mean difference 0.81, confidence interval [0.31, 1.3]).

E. Spectrogram for an individual mutant larva at 6 dpf, where the atrium:ventricle contraction frequency shifted from 1:1 to 2:1 during a two minute recording.

Supplementary figure 5. Auditory sensorimotor processing

A-B. Auditory C-start responsiveness (A) and prepulse inhibition (B) in *cacna1c* wild-type, heterozygote and mutant larvae (N= 48, 82 and 37). Larvae perform two distinct types of auditory C-start response distinguished by latency: short latency C-starts (left) and long-latency C-starts (right). No significant effects of mutation were observed for either mode of response, either at low or high intensity auditory stimuli.

C. Prepulse inhibition of the startle response at 50 ms (left) and 500 ms (right) interstimulus intervals between prepulse and startle inducing stimulus, tested in the same larvae as above. No differences are significant.

Supplementary figure 6. Whole-brain volume and cell density

A. Normalized brain volume in wild-type (N=37), heterozygous (N=18) and mutant (N=12) larvae. Each brain volume was normalized to the mean of the wild-type group. * t-test $p < 0.01$, comparing wild-type and mutants

B. Ratio of the volume of primarily cellular brain areas to neuropil dense areas same larvae as in (A). * t-test $p < 0.001$, comparing wild-type and mutants

C. Mean and confidence interval for difference in cell density for five brain regions in wild-type and mutant larvae (N=16,14 respectively), after DASPEI staining and manual cell counting.

D. Hoechst33342 stained confocal section through the optic tectum in a wild-type and mutant larva.

bioRxiv preprint doi: https://doi.org/10.1101/2025.03.11.642683; this version posted March 14, 2025. The copyright holder for this preprint (which was not certified by peer review) is the author/funder. This article is a US Government work. It is not subject to copyright under 17 USC 105 and is also made available for use under a CC0 license.

E. Volume of the midline cerebellar region that was reduced in TS2 homozygous larvae, in an independent cohort of wild-type (N=24) and sibling heterozygous (N=18) larvae. Mask volume normalized to total brain volume.

F. Mean signal for *sox2* fluorescent *in situ* hybridization at the same coronal section as in Fig. 6D for wild-type (N=10) and mutant (N=9) TS2 larvae. Horizontal confocal scans were co-registered to ZBB, normalized, averaged and re-sliced for coronal view.

Supplementary figure 7. Distribution of post-mitotic neurons

A-B. Mean *tuba:mCardinal* signal within a mask encompassing part of the anterior hindbrain midline area with increased expression in Fig. 7A, in an independent cohort of TS2 wild-type and mutant larvae (N=18, 20 respectively ; t-test, $p < 0.001$; A), and in wild-type and heterozygous larvae (N=24,18 respectively ; t-test, $p = 0.023$; B).

C. Midline *elavl3* fluorescent *in situ* hybridization signal intensity in the anterior hindbrain midline mask, in wild-type and mutant TS2 larvae (N=9 per genotype ; t-test, $p = 0.001$).

D. Meta-analysis of three experiments measuring anterior hindbrain midline *elavl3* signal in TS2 mutants and wild-type larvae. X-axis shows standard mean difference and confidence interval signal intensity between wild-type and mutant larvae in each experiment, and diamond shows a significant overall effect (standard mean difference -0.95, confidence interval [-1.54,-0.36]). There was moderate heterogeneity across experiments ($I^2 = 37.2\%$).

E-G. Subpallial *gad1b:RFP* fluorescence intensity in TS2 wild-type and mutant larvae (E-F, independent cohorts) and wild-type versus heterozygous larvae (G). * t-test, $p < 0.05$.

Supplementary figure 8. Tachycardia and seizure-like activity in heterozygous TS2 larvae at elevated temperature

A-B. Meta-analysis of two experiments measuring cardiac contraction frequency in TS2 wild-type and heterozygous (A) or mutant (B) sibling larvae. X-axis shows mean difference and confidence interval for difference between groups in each experiment. Diamond shows significant combined effect. A: mean difference 51 bpm, confidence interval [36.4,65.8], $p < 0.001$; B: mean difference 41.7 bpm, confidence interval [22.0,61.4], $p < 0.001$.

bioRxiv preprint doi: https://doi.org/10.1101/2025.03.11.642683; this version posted March 14, 2025. The copyright holder for this preprint (which was not certified by peer review) is the author/funder. This article is a US Government work. It is not subject to copyright under 17 USC 105 and is also made available for use under a CC0 license.

C-D. Meta-analysis of three experiments measuring seizure-like behavior in *y680* wild-type and heterozygous (C) or mutant (D) sibling larvae. C: mean difference 0.04 events per minute, confidence interval [0,0.08], p=0.032. D: mean difference 0.18, confidence interval [0.11, 0.25], $p < 0.001$.

Supplementary video 1. Elevated brain activity in mutants

Fluctuating GCaMP fluorescence signal in example wild-type (top) and mutant (bottom) TS2 larvae recorded over 5 min.

Supplementary video 2. Atrium and ventricular contractions

Videos showing atrial (blue outline) and ventricular (orange outline) contractions in wild-type (A) and mutant (B) TS2 larvae, slowed to half speed.

Supplementary Table 1. qPCR primers for measuring exon 8 and 8A usage in cacna1c

Target	Forward primer	Reverse primer	Product size
ef1α (reference)	CTGGAGGCCAGCTCAAACAT	ATCAAGAAGAGTAGTACCGCTAGCATTAC	87
cacna1c exon 8	ATCTGGTTCTGGGTGTGTTG	TCGTCGTCATTCTCAGGGTC	169
cacna1c exon 8A	TGAATGATGCTGTAGGGAATTCC	TTAGATCCTCCTCCAGCTGC	189

Supplementary File 1. Oligonucleotide sequences for HCR probes

Oligonucleotide sequences for probes used to perform HCR fluorescent *in situ* hybridization.

bioRxiv preprint doi: https://doi.org/10.1101/2025.03.11.642683; this version posted March 14, 2025. The copyright holder for this preprint (which was not certified by peer review) is the author/funder. This article is a US Government work. It is not subject to copyright under 17 USC 105 and is also made available for use under a CC0 license.

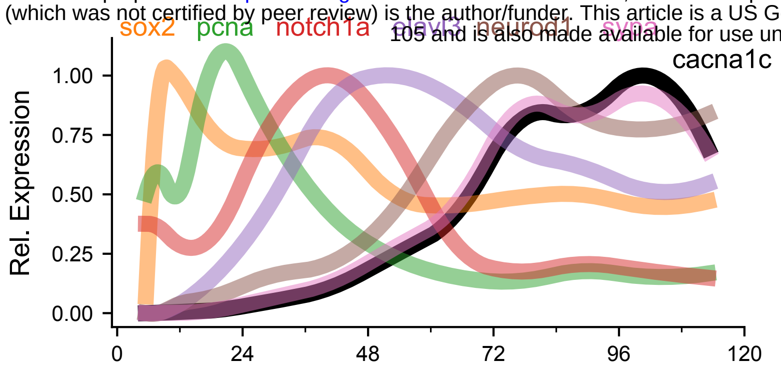

Supplementary Figure 1

bioRxiv preprint doi: https://doi.org/10.1101/2025.03.11.642683; this version posted March 14, 2025. The copyright holder for this preprint (which was not certified by peer review) is the author/funder. This article is a US Government work. It is not subject to copyright under 17 USC 105 and is also made available for use under a CC0 license.

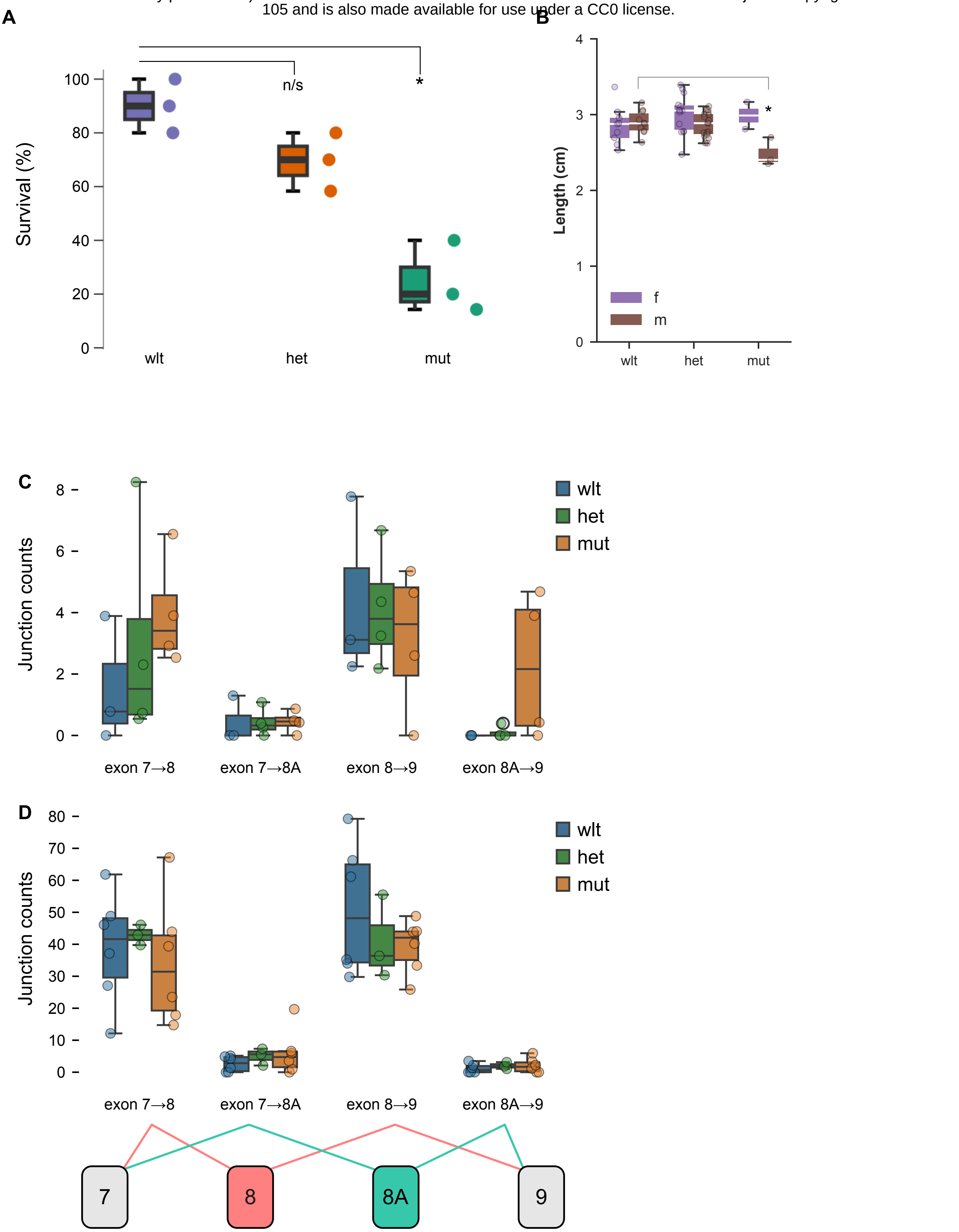

Supplementary Figure 2

bioRxiv preprint doi: https://doi.org/10.1101/2025.03.11.642683; this version posted March 14, 2025. The copyright holder for this preprint (which was not certified by peer review) is the author/funder. This article is a US Government work. It is not subject to copyright under 17 USC 105 and is also made available for use under a CC0 license.

A

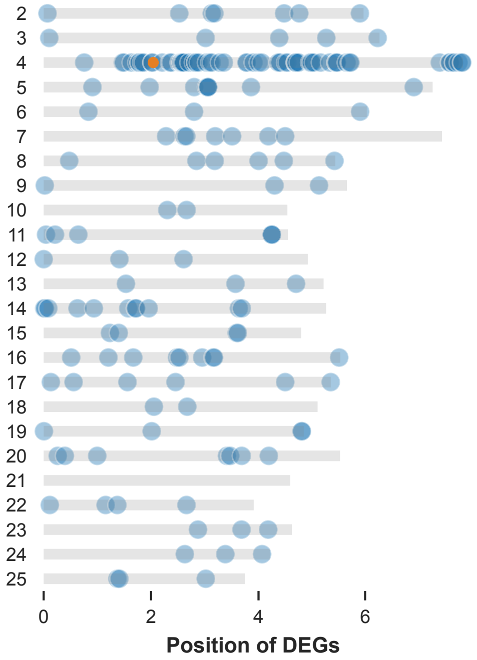

B

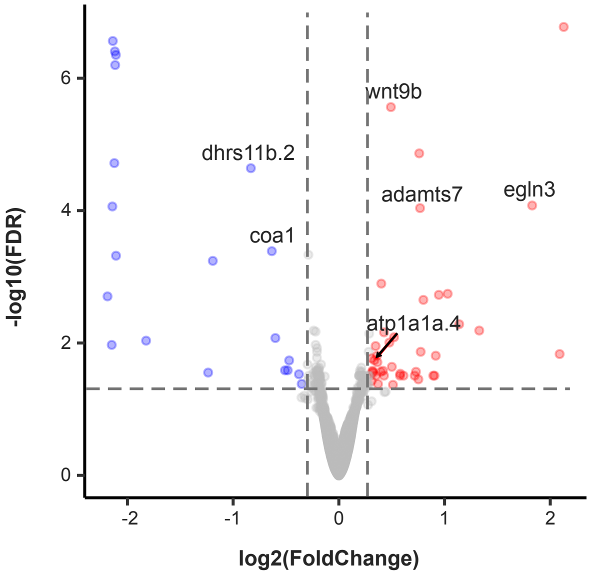

C

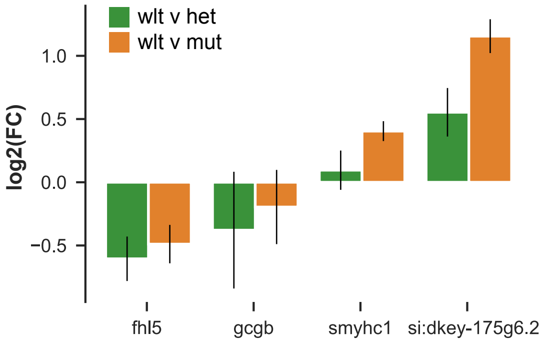

Supplementary Figure 3

bioRxiv preprint doi: https://doi.org/10.1101/2025.03.11.642683; this version posted March 14, 2025. The copyright holder for this preprint (which was not certified by peer review) is the author/funder. This article is a US Government work. It is not subject to copyright under 17 USC 105 and is also made available for use under a CC0 license.

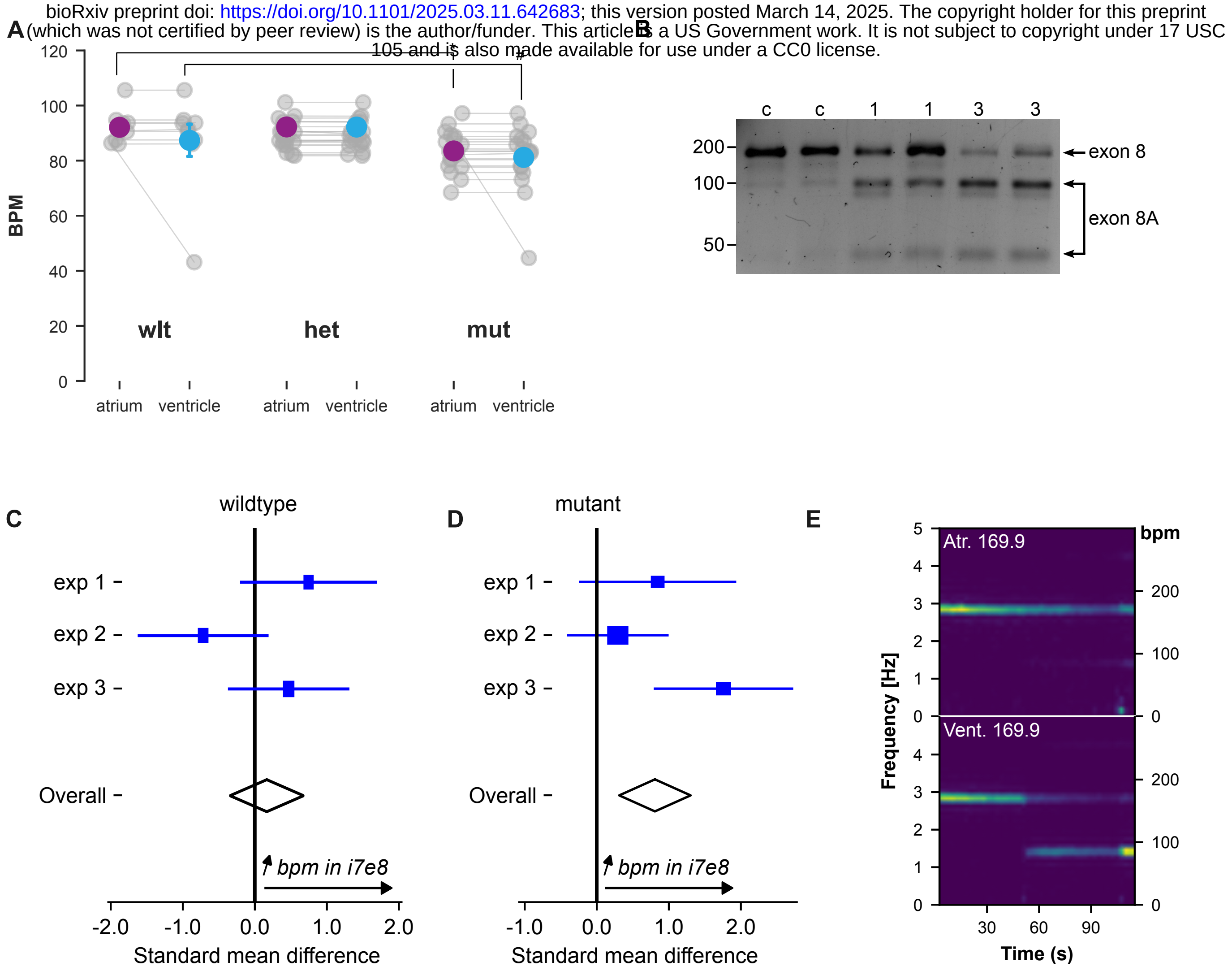

Supplementary Figure 4

bioRxiv preprint doi: https://doi.org/10.1101/2025.03.11.642683; this version posted March 14, 2025. The copyright holder for this preprint (which was not certified by peer review) is the author/funder. This article is a US Government work. It is not subject to copyright under 17 USC 105 and is also made available for use under a CC0 license.

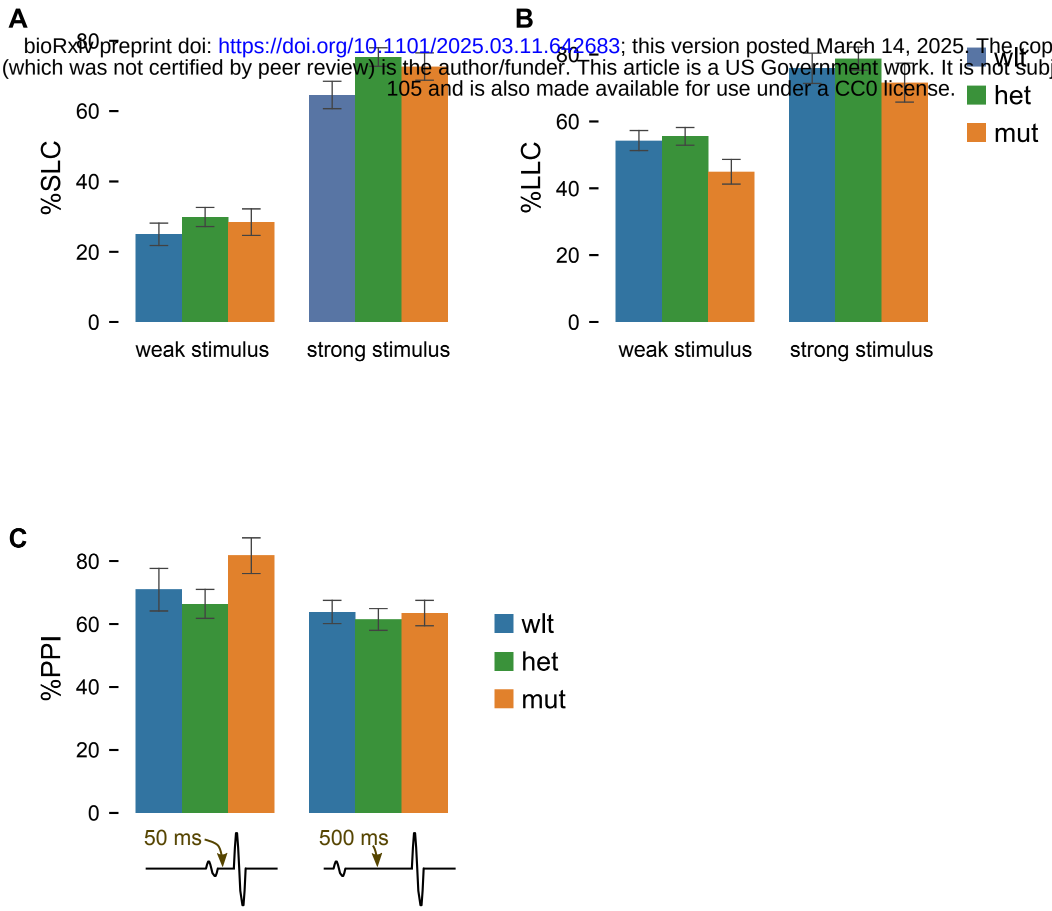

Supplementary Figure 5

bioRxiv preprint doi: https://doi.org/10.1101/2025.03.11.642683; this version posted March 14, 2025. The copyright holder for this preprint (which was not certified by peer review) is the author/funder. This article is a US Government work. It is not subject to copyright under 17 USC 105 and is also made available for use under a CC0 license.

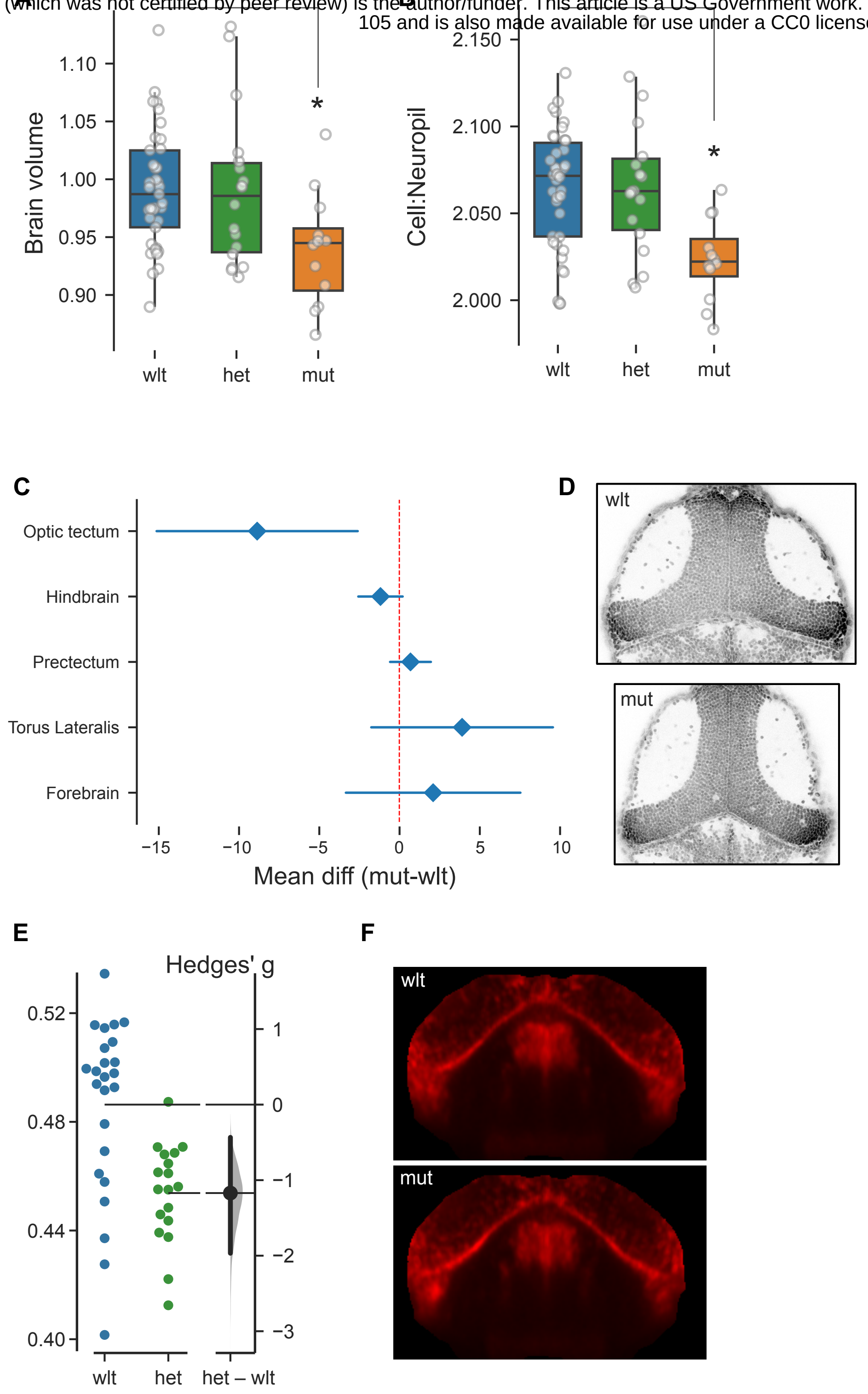

Supplementary Figure 6

bioRxiv preprint doi: https://doi.org/10.1101/2025.03.11.642683; this version posted March 14, 2025. The copyright holder for this preprint (which was not certified by peer review) is the author/funder. This article is a US Government work. It is not subject to copyright under 17 USC 105 and is also made available for use under a CC0 license.

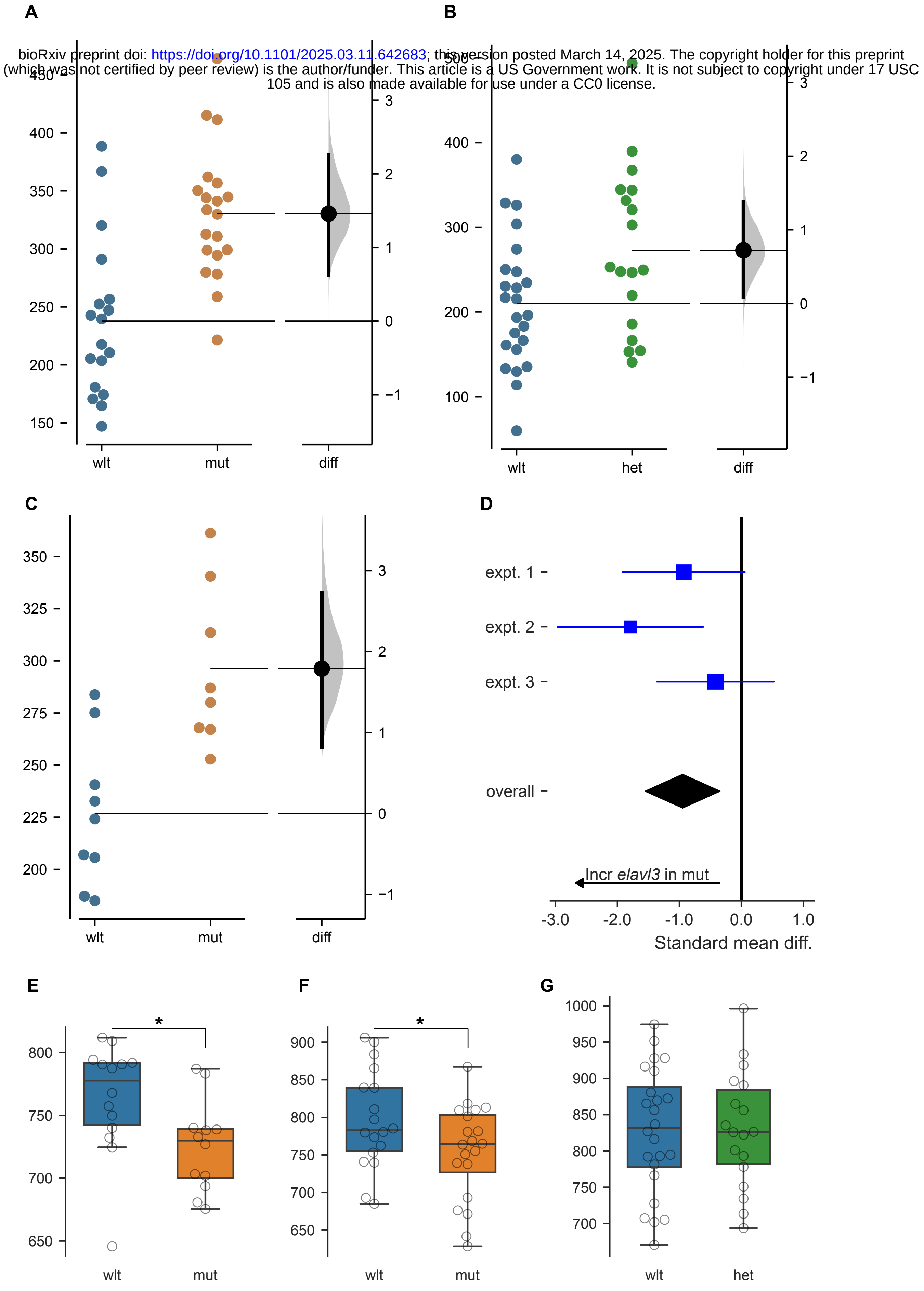

Supplementary Figure 7

bioRxiv preprint doi: https://doi.org/10.1101/2025.03.11.642683; this version posted March 14, 2025. The copyright holder for this preprint (which was not certified by peer review) is the author/funder. This article is a US Government work. It is not subject to copyright under 17 USC 105 and is also made available for use under a CC0 license.

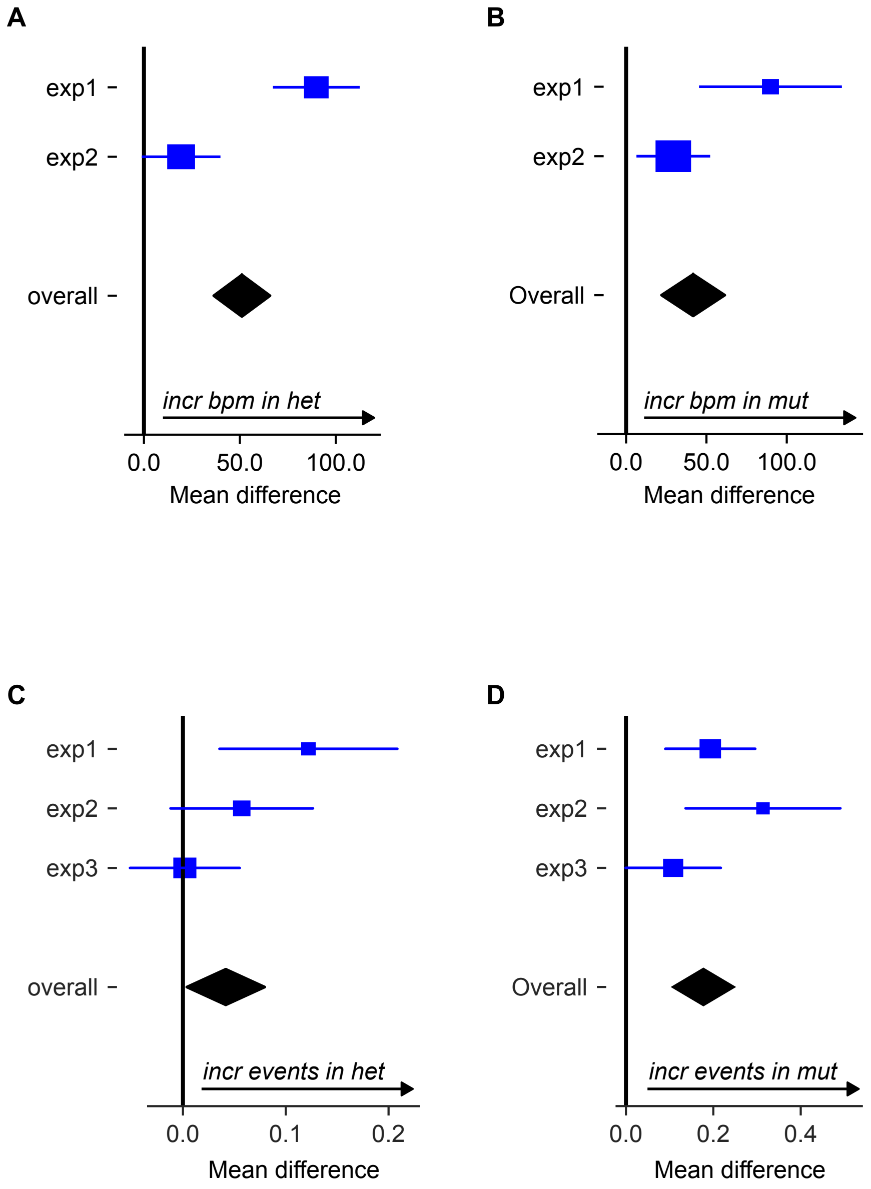

Supplementary Figure 8
